# An Unusual Case of Acute Hemorrhagic Necrotizing Encephalomyelitis in a COVID-19 Patient

**DOI:** 10.7759/cureus.15542

**Published:** 2021-06-09

**Authors:** Moaaz Baghal, Mohamed Ahmed, Mounir Ibrahim, Syed Jafri, Sameh Elias

**Affiliations:** 1 Internal Medicine, Hackensack Meridian Health (HMH) Palisades Medical Center, North Bergen, USA; 2 Neurology, Hackensack Meridian Health (HMH) Palisades Medical Center, North Bergen, USA

**Keywords:** sars-cov-2, acute demyelinating encephalomyelitis, acute hemorrhagic encephalomyelitis, covid-19, case report

## Abstract

Acute disseminated encephalomyelitis, also known as ADEM, is a rare autoimmune demyelinating disease of the central nervous system that has been correlated with viral infections and vaccinations and has a range of presentations, where it can present as a mild neurological dysfunction or more severe manifestations ending in chronic neurological sequelae or even death; therefore, it is considered to be a diagnostic challenge. We present a case of ADEM diagnosed in a previously healthy male patient with a recent infection of SARS-CoV-2 (severe acute respiratory syndrome coronavirus 2). Early diagnosis and management with intravenous immunoglobulins held the key to a good outcome.

## Introduction

The novel coronavirus disease 2019 (COVID-19) has been the new focus of the healthcare system across 217 countries and was labeled as a pandemic in the first quarter of 2020. As of November 2020, it has affected almost 57 million people worldwide [1], and with such a high number of cases, it has unfolded a wide spectrum of disease - it has mainly manifested as a pulmonary illness, but it has also been associated with damage to the heart and kidney, as well as reports of it affecting the central nervous system. Acute disseminated encephalomyelitis (ADEM) is a rare autoimmune disease; it was previously associated more in the pediatric population, but there have been more emerging incidences in the adult population as well, where the diagnosis has mainly been reached through the exclusion of more common diseases. With the emergence of COVID-19 in late 2019, very few cases have been reported where COVID-19 has led to ADEM. We report a case that presented with a rare variant of ADEM, known as acute hemorrhagic necrotizing encephalomyelitis (AHEM).

## Case presentation

A 56-year-old male patient was brought to our hospital with complaints of generalized weakness. He was discharged seven days prior to admission from another hospital for the management of SARS-CoV-2 (severe acute respiratory syndrome coronavirus 2) respiratory illness where he was treated with hydroxychloroquine and discharged on oral amoxicillin. Once discharged home, the patient reported having gradually worsening generalized weakness accompanied by right-sided unilateral loss of vision, slurring of speech, several episodes of nonbilious non-bloody vomiting, and loss of ability to walk without assistance.

He was evaluated in the emergency department, and his Glasgow Coma Scale was 15/15. On examination, he was alert and oriented to place, person, and time; cranial nerves evaluation was unremarkable and there were no signs of meningeal irritation. Examination of the upper and lower extremities showed increased tonicity with brisk deep tendon reflexes. Vital signs on admission showed a blood pressure of 154/85 mm Hg, a pulse of 86 beats per minute, temperature 97.8°F (36.6°C), SPO2 of 95% on room air. On ophthalmic examination, the patient was able to open his eyes spontaneously; the right pupil was 5 mm in diameter, regular, with sluggish reaction to light. Fundoscopic examination of the right eye showed an obliterated disc margin that suggested papillitis.

**Figure 1:**
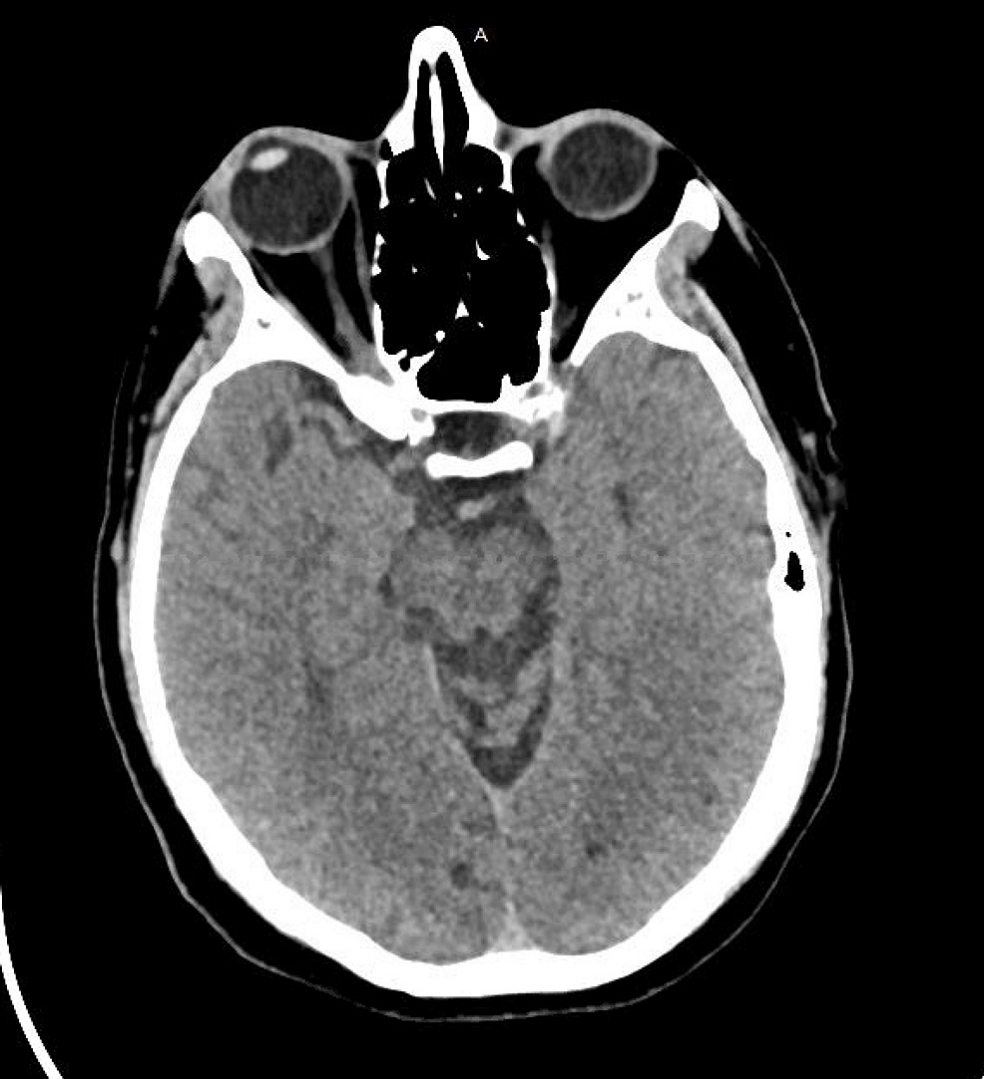
Initial CT of the head without intravenous contrast: no intracranial mass, mass effect, midline shift, or herniation is present. No acute intracranial hemorrhage is present. No findings of an acute territorial infarction are present. No extra-axial collection is present. The ventricular system is normal in size. Brain parenchymal attenuation is normal.

**Figure 2:**
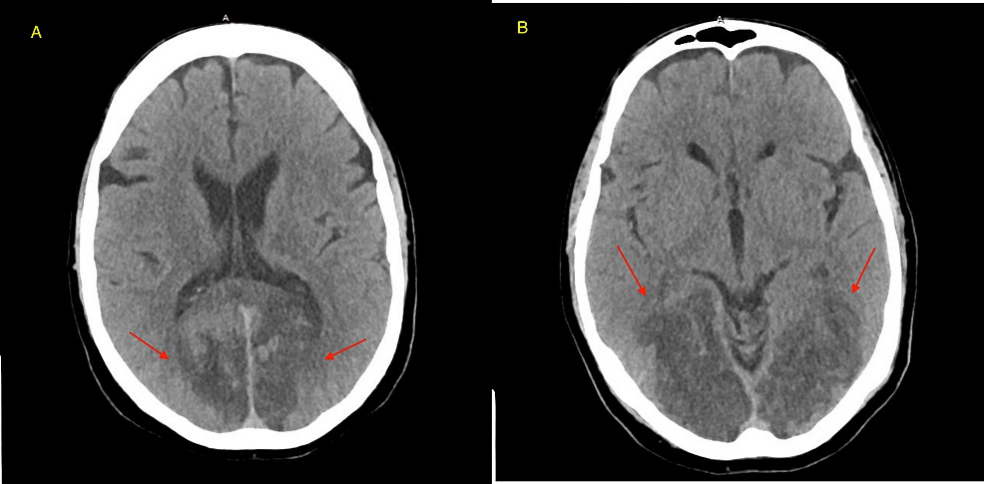
Subsequent CT of the head without IV contrast on day 1 showed ventricles and sulci are normal. No extra-axial fluid collection or subarachnoid hemorrhage is seen. There is no intraparenchymal hematoma or mass. There is loss of gray-white differentiation of the right and left occipital lobes (red arrows in A and B). There is loss of gray-white differentiation of the cerebellum on the right and left, where multiple large acute appearing infarcts are apparent. Dural sinuses are normal in attenuation. Osseous structures are unremarkable. No air-fluid level is noted.

**Figure 3:**
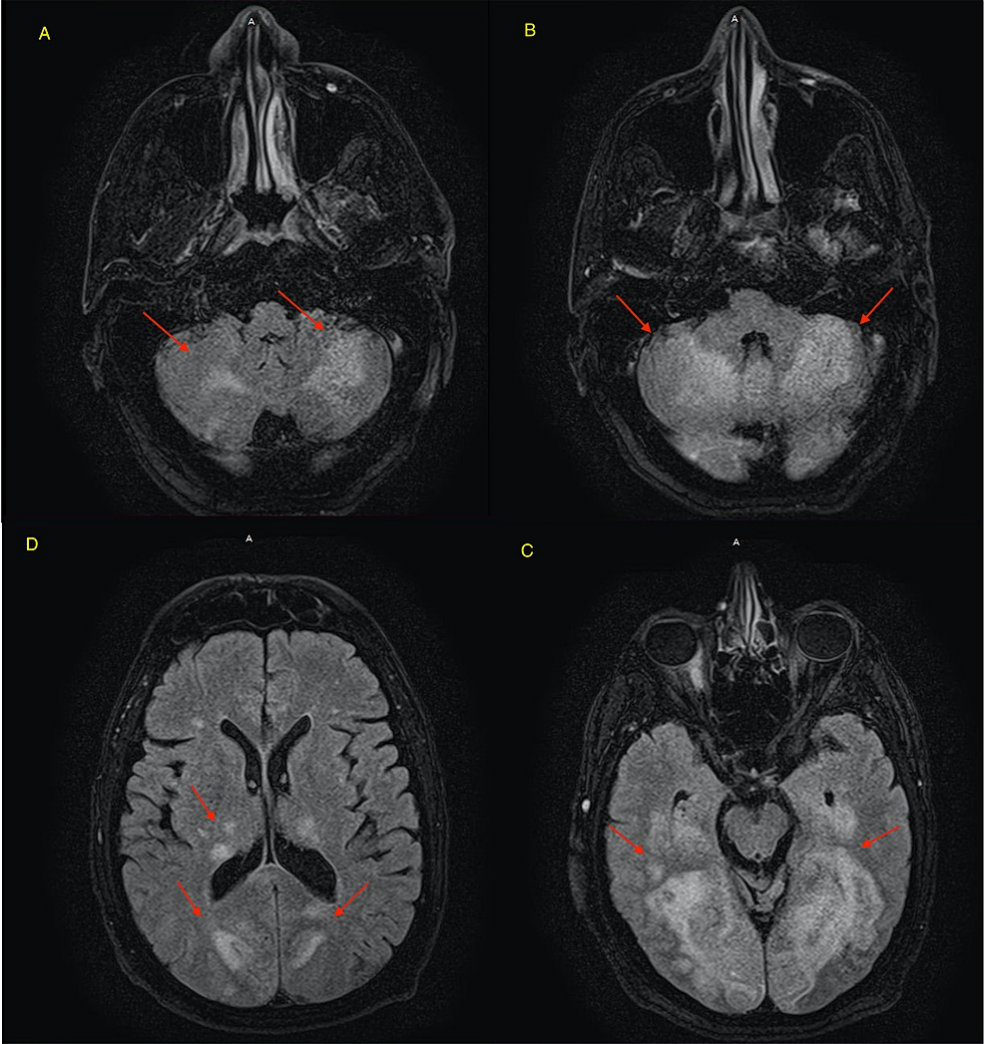
MRI without intravenous contrast on day 7 showed an extensive increased signal intensity in the cerebellum (red arrows in A and B) and in the occipital lobes bilaterally (D and C). There are also abnormal changes in the thalami bilaterally and the splenium of the corpus callosum including a portion of the medial posterior temporal lobes bilaterally. There is associated increased diffusion signal intensity with variable low diffusion signal. These findings are in a posterior circulation distribution bilaterally. Findings are consistent with ischemic changes, which, in a patient with the given history of COVID lung infection, is likely related to sequela of infectious or inflammatory process. Findings related to PRES could be considered though much more extensive than usually expected. PRES, posterior reversible encephalopathy syndrome

On initial blood work, the patient was noted to have leukocytosis (12.9 x 10^3^/uL) with 89% neutrophils, an erythrocyte sedimentation rate of 90 mm/hour, C-reactive protein of 93.6 mg/L, ferritin of 773.2 ng/dL, procalcitonin of 0.13 ng/mL, and vitamin B12 level of 773 ng/mL. Antinuclear antibody and rheumatoid factor were negative. Initial computed tomography (CT) of the head without intravenous (IV) contrast revealed no acute pathology (Figure 1). A chest radiograph revealed multifocal disease with right lower and mid lung consolidation and scattered infiltration of the left lung. Cultures of blood, urine, legionella, and mycoplasma were negative. Subsequent CT of the head without IV contrast on day 1 showed acute appearing right and left occipital lobe infarcts and multiple cerebellar infarcts (Figure 2). The patient was started on aspirin, atorvastatin, and an IV antibiotic (ceftaroline). On day 7, the patient continued to have generalized weakness and visual changes, and magnetic resonance imaging (MRI) showed signs consistent with acute hemorrhagic necrotizing encephalitis (Figure 3). A study of the cerebrospinal fluid (CSF) on day 7 showed the opening pressure to be normal (20 cm CSF). Cultures of the blood and CSF were negative, with only 10 lymphocytes cell/mm^3^, a normal protein level of 300 mg/L, and glucose content of 5.2 mmol/L compared to a random blood glucose level of 7.5 mmol/L. The patient was started on IV immunoglobulins 1 gram daily for three days and then switched to a high dose of oral prednisone of 60 mg/day to be titrated down by 10 mg over six weeks, with discontinuation of aspirin and ceftaroline; heparin for prophylaxis was discontinued as well. Gradual improvement of weakness was noted, as well as improvement in the right visual field. On day 16, the patient was discharged to subacute rehab with a six-week steroid taper.

## Discussion

ADEM has been described to be a monophasic, post-infectious autoimmune disease that has been predominantly reported in healthy young children after a viral infection or vaccinations; however, it has been reported in adult patients as well. The usually suspected viruses associated with ADEM are influenza A virus, influenza B, novel influenza A (H1N1), parainfluenza, varicella, mycoplasma, herpes simplex virus, human herpesvirus-6, human herpesvirus 7, enterovirus, novel reovirus train, rotavirus, and rubella coxsackie A9, with influenza virus and HHV-6 associated infection being the ones most commonly reported [2-4]. With the breakout of the COVID-19 across the globe, different pathologies of the illness have been unraveled, of which a variant of ADEM has been reported known as AHEM, with findings of hemorrhagic lesions in the white matter on imaging and brain biopsy. ADEM was previously reported as associated with COVID-19 by Dixon et al. in a 59-year-old presenting with similar neurological symptoms and findings to our patient 11 days after COVID-19 infection [5].

To reach a diagnosis of ADEM, an exclusion of more common causes must be done that might have a similar presentation, such as multiple sclerosis (MS), infectious meningoencephalitis, neurologic sarcoidosis, progressive multifocal leukoencephalopathy (PML), and vasculitis. CSF analysis did not reveal oligoclonal bands to suggest MS, and normal protein and glucose levels with normal cell differential did not support the finding of infectious meningoencephalitis. The absence of systemic signs of sarcoidosis without findings on brain MRI ruled out sarcoidosis. Vasculitis diagnosis would be supported if brain MRI showed signs of multiple infarcts in multiple vascular territories, which was not evident in this patient's brain MRI. Finally, the diagnosis of progressive PML was ruled out due to the absence of white matter demyelination on brain MRI and due to the more acute course of illness in our patient, unlike the more prolonged onset in PML.

Pathogenesis of the disease is not well understood, but one theory suggests it is to be a cell-mediated response against myelin auto-antigens that share antigenic determinants with a virus, which acts as a trigger for the disease. Talbot et al. reported that molecular mimicry has been implicated in the pathogenesis. They reported a predominance of human coronavirus myelin cross-reactive T-cell lines in MS patients. Virus-myelin T-cell cross-reactivity was also confirmed on the clonal level [6]. There are currently seven coronavirus strains, with SARS-CoV-1, MERS-CoV, and SARS-CoV-2 causing the infection through the upper respiratory airway mainly. SARS-CoV-1 and SARS-CoV-2 enter the body through binding to angiotensin-converting enzyme 2 (ACE-2) [7]. Neurological systems, which also express ACE-2 [8], can get an infection by retrograde trans-synaptic via the peripheral nervous system, and the other route of neurological infection is thought to be by crossing the blood-brain barrier (BBB) through leukocyte migration and sluggish movement of blood across the BBB [9-10]. The most prevalent pathology is related to the reported exaggerated immune response to the Sars-CoV-2 by producing elevated proinflammatory cytokines resembling systemic inflammatory response syndrome, also known as the cytokine storm, with the finding of high levels of CD56+ and natural killer cells during the recovery phase, as reported by Wu et al. [2].

## Conclusions

ADEM is a rare autoimmune inflammatory disease affecting the CNS with an antecedent viral infection, requiring a clinical-radiological approach and a high index suspicion. Clinically, patients presenting with nonspecific and a wide range of neurological findings such as seizures, disturbance of consciousness, and focal neurological dysfunction should be considered for further radiological investigations. Although diagnosis is reached by exclusion, early diagnosis and management with high doses of corticosteroids might be beneficial and lead to a better prognosis. Therefore, we reported our experience in managing this case in the hope to raise the awareness in the medical community of the possible correlation between ADEM and an antecedent COVID-19 infection and a possible further immune-related phenomenon with COVID-19.
